# Catheter-Based Valvular and Structural Interventions in Cancer Patients

**DOI:** 10.1007/s11886-026-02351-x

**Published:** 2026-03-04

**Authors:** Tony Joudi, Ahmad Safdar, Neehal Shukla, Rochell Issa, Zoha Majeed, Alok A. Khorana, Cezar Illescu, Rishi Puri, Rohit Moudgil

**Affiliations:** 1 Community Care Institute, Cleveland Clinic, Cleveland, OH USA; 2 Heart, Vascular and Thoracic Institute, Cleveland Clinic, Cleveland, OH USA; 3 Taussig Cancer Center, Cleveland Clinic, Cleveland, OH USA; 4 Department of Cardiology, MD Anderson Cancer Center, University of Texas, Houston, TX USA

**Keywords:** Cardio-oncology, Transcatheter valve replacement, Mitral valve repair, Structural heart disease, Patent foramen ovale, Left atrial appendage occlusion, Bleeding risk

## Abstract

**Purpose of Review:**

This review examines the evolving role of catheter‐based valvular and structural interventions in cancer patients. We sought to answer whether minimally invasive approaches, including transcatheter aortic valve replacement, mitral valve repair, left atrial appendage occlusion, and patent foramen ovale closure, provide safe and effective treatments in high‐risk oncologic populations.

**Recent Findings:**

Emerging research indicates that these interventions yield comparable short‐term outcomes in cancer and non‐cancer patients, with reduced procedural complications, lower bleeding risks, and improved recovery times. Studies also suggest that careful patient selection and tailored antithrombotic management are critical, as long‐term survival is affected by the underlying malignancy.

**Summary:**

Our review concludes that catheter‐based interventions offer significant benefits in managing cardiovascular complications in cancer patients. Future investigations should focus on refining selection criteria, optimizing perioperative care, and evaluating long‐term outcomes to enhance interdisciplinary treatment strategies. These findings pave the way for improved care via a tailored approach to patients with both cancer and cardiovascular disease.

## Introduction

Cardio-oncology is an expanding field, as cardiovascular disease and cancer remain the leading causes of morbidity and mortality worldwide [1]. Advances in cancer therapies have increased survival rates, leading to a growing population of cancer patients who often face long-term cardiovascular complications, including valvular heart disease (VHD). The management of VHD in this population is uniquely complex, as cancer patients often face heightened risks of bleeding, thrombosis, and infections, compounded by the cardiotoxic effects of oncologic therapies such as chemotherapy and radiation.

In recent years, there has been a growing interest in transitioning from surgical towards transcatheter approaches in cancer patients. This shift is driven by the need for less invasive procedures with lower perioperative risks, favorable outcomes, and the ability to continue essential cancer treatments with minimal disruption [2, 3]. Since the introduction of transcatheter interventions, cancer patients have been underrepresented in clinical trials. The initial PARTNER (Placement of Aortic Transcatheter Valve Trial) included only 6.5% of participants with a cancer diagnosis [4]. In 2019, Landes et al. was the first group that highlighted outcomes of transcatheter aortic valve replacement (TAVR) in cancer patients [5]. Nonetheless, data regarding periprocedural complications and short- and long-term survival in patients with cancer treated with catheter-based valvular interventions remains scarce.

**Fig. 1:**
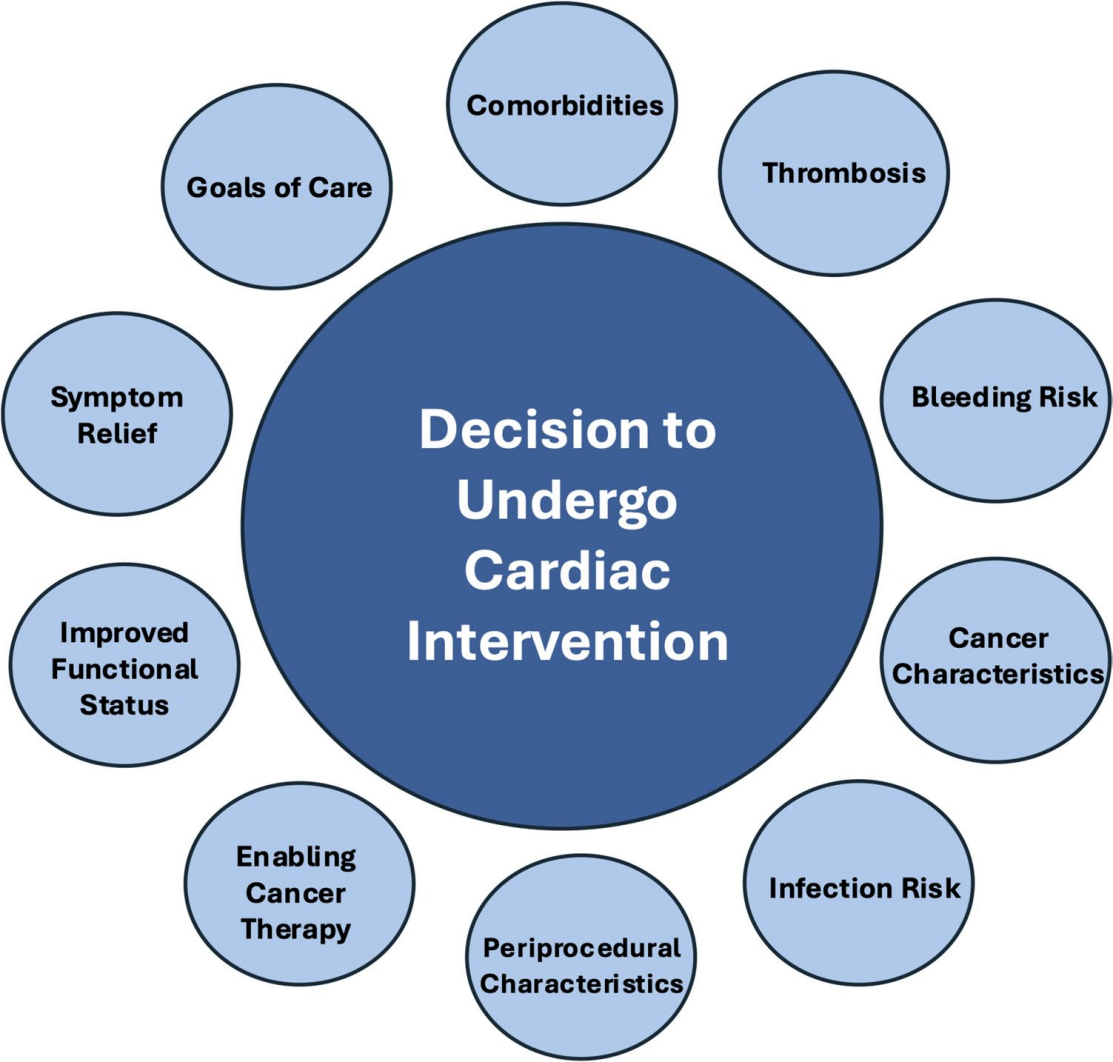
Elements of the decision to proceed with cardiac interventions

**Fig. 2:**
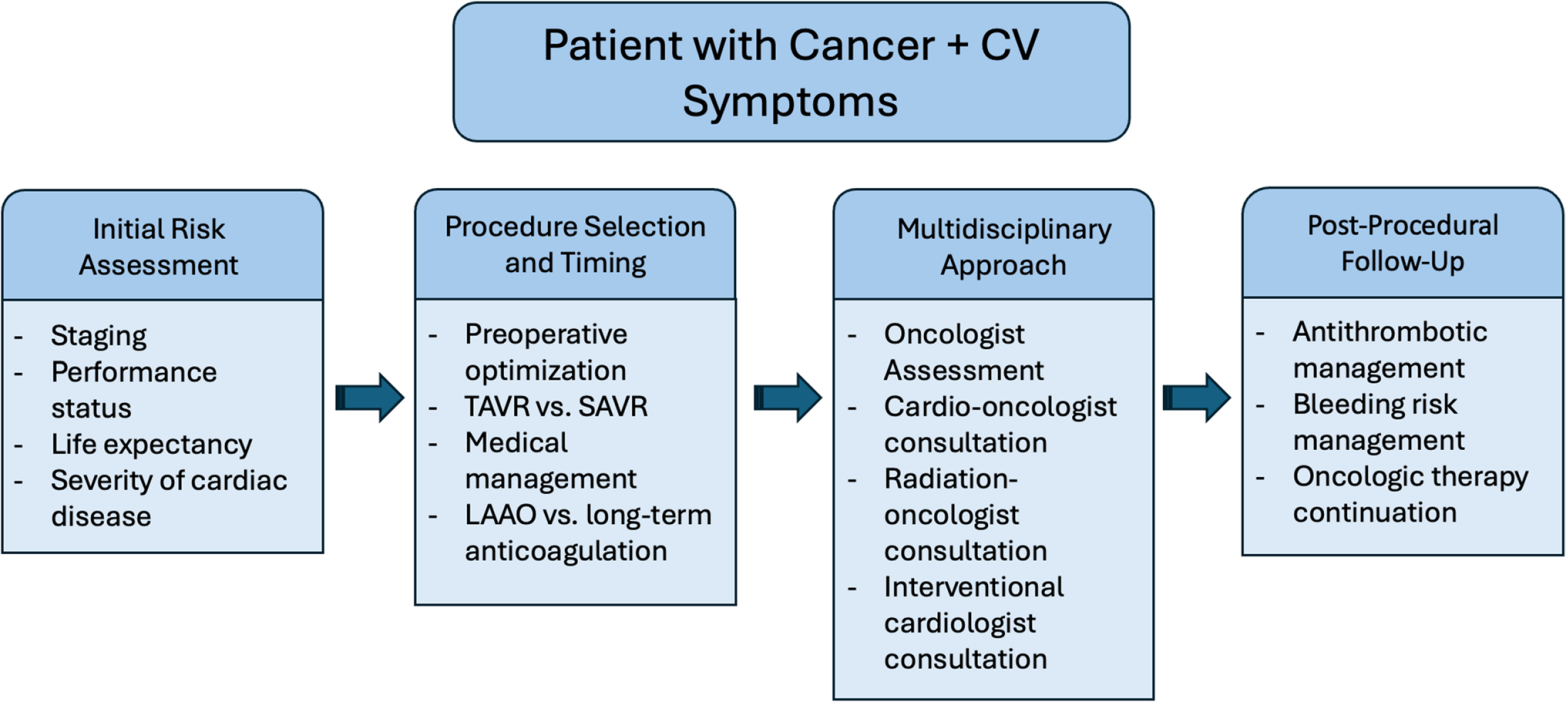
A multidisciplinary flowchart for evaluating and treating patients with active or recent malignancy who require valvular or coronary interventions. Each step underscores collaborative decision making among oncologists, cardiologists, and interventionalists. Key: TAVR, transcatheter aortic valve replacement; SAVR, surgical aortic valve replacement; LAAO, left atrial appendage occlusion

This review explores the current evidence on structural cardiac interventions in the management of cancer patients, emphasizing their role in management and the unique challenges posed by this population (Fig. 1). It also examines critical issues such as radiation-induced cardiac damage and bleeding risks, underscoring the importance of a multidisciplinary care team–including oncologists, cardiologists, interventional cardiologists, and cardiac surgeons—to develop personalized strategies that optimize patient outcomes (Fig. 2).

## Transcatheter Aortic Valve Replacement

Aortic stenosis (AS) is the most prevalent valvular heart disease globally. The PARTNER 1 A medical arm cohort demonstrated a 50% mortality rate at 2–3 years, while the RECOVERY (Randomized Comparison of Early Surgery versus Conventional Treatment in Very Severe Aortic Stenosis) trial reported a cumulative incidence of all-cause mortality of 32% at 8 years in the conservative-care group—an ominous prognosis that rivals or surpasses many types and stages of cancer [1–3]. In cancer patients, AS poses a significant challenge as it can hinder optimal cancer treatment, such as high-risk surgeries or tolerance to oncologic drugs. The European Society of Cardiology recommends afterload reduction with angiotensin-converting enzyme inhibitors or angiotensin II receptor antagonists to mitigate left ventricular dysfunction and heart failure from cancer therapies [4]. However, in AS patients, effective afterload reduction can typically be best achieved through aortic valve intervention. Historically, severe AS in cancer patients often led to prioritizing cancer treatment over surgical aortic valve replacement (SAVR). Those who underwent SAVR demonstrated significantly better survival; however, SAVR was associated with increased perioperative mortality and morbidity when comparing patients with and without cancer [6, 7]. The prognosis of cancers has significantly improved over the past 20 years due to advancements in early detection, treatment modalities, and overall cancer management. Transcatheter aortic valve replacement (TAVR) offers a less invasive alternative to SAVR, reducing the risks of bleeding and venous thromboembolism (VTE) while minimizing delays in resuming cancer treatment due to faster recovery times [8–10]. The CoreValve U.S. Pivotal Trial demonstrated that TAVR with the self-expanding prosthetic valve resulted in significantly lower all-cause mortality at 1 year compared to SAVR (14.2% vs. 19.1%, *p* = 0.04) in high-risk patients. The advantage of self-expandable prosthetic valves extended to stroke outcomes, with a lower incidence of major stroke at 1 year (8.8% for self-expandable prosthetic valves vs. 12.6% for SAVR) [11]. While TAVR has emerged as a promising intervention to treat aortic valve disease in a myriad of patient profiles, major randomized controlled trials (RCTs) have excluded cancer patients with a life expectancy < 2 years [12, 13].

Landes et al., using data from the TOP-AS (TAVR in Oncology Patients with severe AS) registry, reported comparable 30-day mortality rates between cancer and non-cancer patients undergoing TAVR. However, 1-year mortality was significantly higher in cancer patients (15% vs. 9%; *p* < 0.001), largely driven by cancer progression [5]. Notably, 85% of cancer patients were alive at 1 year after the procedure, which exceeds conventional post-TAVR benchmarks, such as the 75% 1-year survival rate reported in the PARTNER high-risk trial and the FRANCE 2 (French Aortic National CoreValve and Edwards) registry [14, 15]. Furthermore, these patients had symptomatic benefit with regard to New York Heart Association (NYHA) functional class, albeit less pronounced compared with non-cancer patients. A meta-analysis of 13 studies looking at TAVR outcomes in patients with and without cancer by Murphy et al. found no significant differences in short-term mortality (relative risk [RR] 0.61, 95% confidence interval [CI] 0.36–1.01; *p* = 0.06) and long-term all-cause mortality (RR 1.24, 95% CI 0.95–1.63; *p* = 0.11) [16]. Similarly, a meta-analysis by Marmagkiolis et. al reported lower short-term mortality in cancer patients of 2.4% compared with 3.3% in the control group (odds ratio: 0.72, 95% CI 0.63–0.82, *p *< 0.0001) [16]. Ahsan et. al’s metanalysis of 15 studies showed no difference in all-cause mortality [16, 17]. These findings suggest that while TAVR provides comparable short-term outcomes for cancer patients, long-term survival remains influenced by the complexities of cancer progression, underscoring the importance of tailored treatment strategies that integrate both oncologic and cardiovascular care.

Given the minimally-invasive nature of TAVR, it is not surprising that TAVR in cancer patients is associated with lower risks of postprocedural acute kidney injury (AKI) and major bleeding compared to SAVR [9, 10]. Moreover, performing surgical procedures in patients with cancer presents challenges, particularly regarding potential perioperative complications. For instance, infections are a notable concern following SAVR in individuals with leukemia or compromised immune systems [7]. Chemotherapy can hamper the healing process of sternotomy wounds leading to increased risk of infection. Therefore, these life-saving cancer therapies need to be suspended if SAVR is used as an option. Watanabe et al. analyzed a TAVR database from eight Japanese centers and reported no significant differences in the rates of major vascular complications (4.3% in cancer patients vs. 7.5% in non-cancer patients, *p* = 0.24), life-threatening bleeding (2.1% vs. 7.1%, *p* = 0.15), and major bleeding (8.5% vs. 13%, *p* = 0.38) [19]. Ullah et al. evaluated patients using the Nationwide Inpatient Sample database and a subset of those were propensity-score matched with and without cancer to calculate adjusted odds ratio for MACE after TAVR and found that risk was largely contingent on cancer type. Patients with prostate, breast, lung, and renal cancers had similar or lower risks of mortality and MACE when comparing TAVR to SAVR in that study (lung cancer aOR 0.65, 95% CI 0.43–0.97; prostate cancer aOR 0.79, 95% CI 0.66–0.96) [18]. Building on these tumor-specific trends, in their “Heart Valve Team conundrum” editorial, Faggiano et al. propose a practical framework for managing severe AS in patients with malignancy, emphasizing a post-procedural life-expectancy threshold ≥ 1 year to avoid futility and the need for multidisciplinary prognosis estimation with oncology; timing considerations—when urgent cancer therapy cannot be delayed, TAVR is favored because it typically permits resumption of treatment in approximately 2 weeks rather than 2 months after SAVR; and procedure selection that incorporates remission status and features tipping the balance toward TAVR (e.g., thrombocytopenia/anemia, prior chest radiation, porcelain aorta, prior cardiac surgery, frailty). They also note that tumor site may influence strategy and provide an algorithm operationalizing these steps. This editorial perspective complements tumor-specific outcomes and supports individualized, heart-team decision-making in this population [19].

Aikawa et al. assessed the Nationwide Readmissions Database for TAVR and found patients with active cancer were at increased risk of bleeding requiring transfusion during the index hospitalization and readmission at 30, 90, and 180 days after TAVR compared to those without cancer [20]. This aligns with previous studies that have found increased bleeding events and need for transfusion in cancer patients [5, 21]. These findings underscore bleeding vulnerability in active cancer following TAVR and reinforce individualized modality selection rather than a universal preference for SAVR or TAVR. Additionally, chemotherapy and radiation play significant roles in these complications, as they have been associated with increased risk of hemolysis, blood loss, bone marrow failure, infections, and inflammatory responses.

Cancer patients often have thrombocytopenia, and interventionalists must balance the risk of thromboembolic events, such as hypo-attenuated leaflet thickening (HALT), against the risk of bleeding. HALT is associated with increased transvalvular gradients, and the PARTNER 3 trial demonstrated a potential link between HALT and cerebrovascular events [22, 23]. HALT occurs more frequently in transcatheter than in surgical bioprosthetic valves [24]. Guidelines for antithrombotic therapy post-TAVR vary among national societies due to limited robust evidence. The ACC/AHA recommends dual antiplatelet therapy (DAPT) for 3–6 months, followed by lifelong single antiplatelet therapy (SAPT), with VKA as an option for low-bleeding-risk patients [25]. The Canadian Cardiovascular Society (CCS) favors a minimalist approach, recommending SAPT with aspirin unless another indication exists [26]. Multiple meta-analyses comparing SAPT and DAPT post-TAVR have shown no significant difference in ischemic events or stroke at both short-term (30 days) and long-term (6–12 months) follow-up [27–29]. While risks of bleeding have not been specifically studied in patients with thrombocytopenia post-TAVR, in cancer patients with severe thrombocytopenia (platelet count < 50,000 cells per uL) and acute myocardial infarction, aspirin has been associated with improved survival without a significant increase in major bleeding events [30]. A thoughtful approach to antithrombotic management in thrombocytopenic patients undergoing TAVR is essential, balancing thromboembolic risk with bleeding concerns, while further research is needed to guide optimal therapy in this high-risk population.

### Transcatheter Mitral Valve Repair

Mitral regurgitation (MR) is one of the most prevalent valvular heart diseases. When left untreated, severe MR can worsen heart failure symptoms, significantly reduce functional capacity, compromise quality of life, and ultimately increase the risk of mortality. Transcatheter mitral valve repair (TMVr), particularly with the mitral transcatheter edge-to-edge repair system, has emerged as an alternative to surgery for inoperable patients with degenerative MR or severe symptomatic functional MR unresponsive to guideline-directed medical therapy [24]. Current guidelines recommend mitral transcatheter edge-to-edge repair systems for patients with an expected survival of more than 1 year, but estimating life expectancy in cancer patients is challenging. Symptomatic MR can hinder effective cancer management, making these interventions a potential option to enable life-prolonging cancer therapies. Cancer survivorship is often associated with accelerated functional decline and reduced quality of life, making these measurable improvements through TMVr especially impactful.

Limited data exist on the outcomes of cancer patients undergoing TMVr. In a single-center study, Oner et al. reported that cancer patients undergoing TMVr had more than twice the mortality risk compared to non-cancer patients, with an estimated 1-year mortality rate of 56%. Of note, this study included only 19 cancer patients and was conducted during the early adoption of TMVr. Furthermore, it included patients with higher overall risk profiles with greater burden of comorbidities among cancer patients—all of which are known predictors of increased mortality [22]. In a larger cohort study that included 82 cancer patients, Tabata et al. observed a 1-year mortality rate of 20% in cancer patients, more than double that of non-cancer patients. The risk persisted even after adjusting for confounding factors [23]. Similarly, Kalkan et al., in their analysis of 143 patients, found comparable 1-year mortality rates. Notably, they found that TMVr significantly improved symptoms, physical capacity, and quality of life in cancer patients, including those with active disease, with benefits similar to those seen in non-cancer patients. The largest study to date was done by Bansal et al., who utilized a nationwide readmission database and reported comparable short-term survival and major in-hospital outcomes between cancer and non-cancer patients undergoing mitral transcatheter edge-to-edge repair. Cancer patients who underwent TMVr faced significantly higher rates of 30-day readmissions, heart failure hospitalizations, and blood transfusion requirements than non-cancer patients, reflecting the unique challenges posed by this population [25]. Another national database study by Guha et al. found no difference in hospital mortality or safety profile with the use of TMVr or surgical mitral valve procedures (SMVP), based on the presence or absence of cancer. TMV repair may be associated with an even greater safety profile compared with SMVP because of increased episodes of major bleeding associated with SMVP in cancer [13]. This may be explained by the observation that cancer patients usually tend towards increased bleeding following a major surgical procedure owing to their inflamed and hypercoagulable nature, as well as persistent thrombocytopenia. Nonetheless, larger prospective multicenter studies would strengthen the level of evidence for TMVr in cancer patients.

### Left Atrial Appendage Occlusion (LAAO)

Atrial fibrillation (AF) remains a significant contributor to cardioembolic stroke, largely due to thrombus formation in the left atrial appendage (LAA). Standard management includes oral anticoagulation, now commonly employing direct oral anticoagulants (DOACs). However, these agents carry a risk of bleeding, which can be especially relevant in cancer patients who may already have thrombocytopenia or other hematologic abnormalities.

A growing body of evidence supports LAA occlusion (LAAO) in individuals with AF who cannot tolerate chronic anticoagulation. The LAAOS III trial showed that surgical ligation of the LAA during cardiac surgery reduced stroke by 33% compared with leaving the LAA intact [31]. Less invasive approaches, such as a left atrial appendage occlusive device, have also demonstrated favorable outcomes in randomized trials (PROTECT AF and PREVAIL), with reductions in hemorrhagic stroke, cardiovascular mortality, and nonprocedural bleeding compared to warfarin[32, 33].

In patients with cancer, the hypercoagulable state and frequent drug-drug interactions elevate both thrombotic and bleeding risks, making LAAO an attractive alternative. Recently, we performed a real-world data from a TriNetX analysis, a health research network accessing data from over 100 million patients globally, which further underscores the potential benefits of LAAO in cancer patients with AF. In this study, 294 cancer patients with AF were identified, of whom 147 underwent LAAO. Over a 5-year follow-up period, those who received LAAO had significantly lower mortality and fewer major bleeding events compared to those managed without LAAO. These findings suggest that LAAO is a beneficial intervention for improving survival and reducing bleeding complications in this high-risk patient population. By avoiding or minimizing the duration of systemic anticoagulation, LAAO provides a promising mechanical strategy to reduce thromboembolic events while simultaneously lowering the risk of serious bleeding [34].

### PFO/ASD Closure

Patent foramen ovale (PFO) is a remnant of fetal circulation in which a flap-like opening between the atria persists into adulthood, reported in approximately 25% of individuals, although estimates range from 10 to 35% [35, 36]. While many remain asymptomatic, a PFO can permit venous thrombi to cross into systemic circulation (paradoxical embolism), posing a risk of cryptogenic stroke. Indeed, in patients presenting with cryptogenic stroke, nearly half may have an underlying PFO [37].

Historically, treatment options for PFO included medical therapy with antiplatelet agents, anticoagulants, or surgical closure. Over the past few years, novel percutaneous closure devices have been developed, delivering occluders via catheter-based systems that straddle and seal the interatrial defect [38]. Søndergaard and colleagues conducted a multinational trial examining antiplatelet therapy alone versus antiplatelet therapy plus PFO closure; those who underwent closure had significantly lower rates of both clinical ischemic stroke (1.4% vs. 5.4%, *p* = 0.002) and silent brain infarctions on MRI over a median 3.2-year follow-up [38]. Notably, PFO devices themselves are thrombogenic and typically require some duration of anticoagulation and single anti-platelet therapy (SAPT). There is no current consensus on the overall duration of agents for anti-platelet and anti-thrombotic agents in patients without cancer and given increased theoretical risks of device-related thrombosis in cancer patients, an individualized approach is necessary for this group [39].

Data specifically evaluating PFO in cancer patients are limited. Nonetheless, hypercoagulability in malignancy increases the risk of paradoxical emboli, highlighting the potential significance of PFO detection. Case reports have documented cryptogenic stroke as the initial presentation of occult metastatic disease, implicating a PFO in stroke pathogenesis [40]. By contrast, some studies have shown fewer clear associations; one analysis found that while 4.6% of stroke patients had active cancer, only 23% of those with cancer had a PFO compared to 36% without cancer [41]. Further research is needed to elucidate whether routine PFO screening in cancer patients is warranted, particularly in the context of known malignancy-associated coagulopathies and potential stroke mechanisms[42].

### Specific Considerations for Cancer Patients Undergoing Catheter-Based Cardiac Procedures

Patients with cancer present a constellation of cardiovascular risks that may affect the safety and efficacy of catheter-based cardiac procedures. Before, during, and after interventions such as TAVR, TMVr, or LAAO, interventionalists must consider the unique interplay between malignancy-related thrombosis, bleeding tendencies, prior cardiotoxic therapy, and radiation-associated anatomic changes. The following subsections outline how these factors influence procedural planning, peri-procedural management, and long-term outcomes. These multidisciplinary assessments align with the 2022 European Society of Cardiology (ESC) Cardio-Oncology Guidelines, which emphasize pre-procedural risk stratification, careful evaluation of cancer-therapy–related cardiotoxicity, and individualized antithrombotic management before and after interventional procedures [43].

### Stroke

Among interventional candidates, stroke represents a serious complication driven by hypercoagulability related to tumor-produced procoagulants, inflammatory cytokines, and disruptions in normal hemostasis [44, 45]. Although the pulmonary circulation typically filters venous thrombi, a PFO or ASD can enable a right-to-left shunt, resulting in paradoxical embolic events. In addition, AF is increasingly recognized in patients with malignancies, further raising the risk of thromboembolic stroke by promoting clot formation in the LAA [46, 47]. This is in the background of the fact that cancer itself is a very thrombotic entity as illustrated by prophylactic treatments of VTE in some of the cancer patients such as GI and GU malignancies. Even a risk scoring system (Khorana score) has been devised to treat high risk patients [48, 49]. This elevated thromboembolic risk has direct implications for transcatheter interventions, where periprocedural anticoagulation and cerebral protection strategies may need modification.

A study by Letteri et al. looked at acute stroke patients with cancer and explored the use of mechanical thrombectomy for cancer patients given concerns associated with hemorrhagic transformation in cancer patients when using thrombolytic therapy options. The Italian Registry of Endovascular Treatment in Acute Stroke provided data for 4598 stroke patients. In this cohort, outcomes of endovascular therapy (EVT) were compared in patients with and without comorbid cancer and showed comparable bleeding risk between both groups (8.2% vs 6.9%). They also found that certain cancer types (e.g., breast) were associated with lower 3-month mortality, underscoring the importance of risk-stratification [50].

Even in long-term cancer survivors, the risk of stroke remains heightened. A meta-analysis involving over 10 million participants showed a 66% higher relative risk of stroke in individuals with a history of cancer compared to those without [42]. Further, some registry data suggest that active cancer may be associated with multifocal embolic patterns and increased stroke severity [48]. Because cancer therapies can also accelerate atherosclerosis or potentiate vascular injury, thorough cardiovascular evaluation is essential for patients diagnosed with cancer—particularly those displaying cryptogenic stroke or new AF.

### Bleeding

Bleeding risk is a key determinant of procedural safety in transcatheter valve or appendage occlusion procedures. Deregulation of the coagulation cascade in cancer can lead to significant bleeding concerns—accounting for 10% in the overall population of patients with cancer, increasing to 30% in those with blood cancers. The spectrum of bleeding can range from clinically insignificant bleeding and chronic anemia to overt hemorrhagic shock—and can be a direct result of the cancer itself or the treatments options available for therapy—including chemotherapy, radiotherapy, or newer targeted therapies (immunotherapy, monoclonal antibodies) [51–54]. Especially, patients with cancer receiving anticoagulation, they have a two- to threefold increase in major bleeding risk compared with anticoagulated patients without cancer [55, 56]. Bleeding from unresected primary tumors, particularly GI tract, genitourinary tract, and gynecologic malignancies, is common while on anticoagulation. Factors that increase the risk of venous thromboembolism (VTE) can also increase the bleeding risk with anticoagulation, including tumor site of origin [57], advanced/metastatic disease [58], cytotoxic agents, radiation therapy, and surgery [59].

Khorana and his colleagues examined the incidence of bleeding in patients who received anticoagulation comparing cancer versus non-cancer patients [60]. Data were obtained from Explorys (IBM Watson, Inc.), which pools data from multiple US healthcare organizations. Cohorts of patients were created to compare bleeding events between cancer and non-cancer patients treated with anticoagulation within 6 months of starting anticoagulation [60]. The cohort comprised 3,283,140 cancer patients, of whom 435,140 (13.3%) received anticoagulation within 6 months of their cancer diagnosis. Bleeding incidence was higher in cancer vs non-cancer patients across all anticoagulants studied [60]. Furthermore, patients with gastrointestinal malignancies had a higher incidence of bleeding compared to other tumors across all anticoagulants [60]. Other factors associated with increased risk of bleeding included metastatic disease, chronic kidney disease, and thrombocytopenia. Recognizing baseline anemia and thrombocytopenia is essential before device implantation or vascular access.

Recently, a multicenter clinical trial (SELECT-D) was conducted to directly compare rivaroxaban against low molecular weight heparin in cancer patients. In this randomized, open-label, pilot trial in the UK, patients with active cancer who had symptomatic pulmonary embolism (PE), incidental PE, or symptomatic lower-extremity proximal deep vein thrombosis (DVT) were recruited [61]. Patients were allocated to dalteparin (200 IU/kg daily during month 1, then 150 IU/kg daily for months 2–6) or rivaroxaban (15 mg twice daily for 3 weeks, then 20 mg once daily for a total of 6 months) [61]. The primary outcome was VTE recurrence over 6 months. Although the VTE risk was low in rivaroxaban vs dalteparin, the 6-month cumulative rate of major bleeding was 4% (95% CI, 2% to 8%) for dalteparin and 6% (95% CI, 3% to 11%) for rivaroxaban (HR, 1.83; 95% CI, 0.68 to 4.96) [61]. Corresponding rates of clinically relevant non-major bleeding (CRNMB) were 4% (95% CI, 2% to 9%) and 13% (95% CI, 9% to 19%), respectively (HR, 3.76; 95% CI, 1.63 to 8.69), with combined risk of bleed of 11% at 6 months in rivaroxaban [61]. This is illuminating as the EINSTEIN investigators who examined the incidence of VTE and bleeding in a non-cancer patient population (5% patients had active cancer) showed a combined bleeding risk of 8.1% (fatal or CRNMB) after 12 months with only 6% at 6 months with rivaroxaban [62]. Similarly, CARAVAGGIO trial was conducted with apixaban and compared with dalteparin in patients with VTE and cancer [63]. Clinically relevant non-major bleeding occurred in 52 patients (9.0%) with apixaban and in 35 patients (6.0%) with dalteparin (hazard ratio, 1.42; 95% CI, 0.88 to 2.30); major or clinically relevant non-major bleeding occurred in 70 patients (12.2%) and in 56 patients (9.7%), respectively (hazard ratio, 1.16; 95% CI, 0.77 to 1.75), at 6 months [63]. The rates of bleeding were considerably higher when compared to data from AMPLIFY study which showed the composite outcome of major bleeding and clinically relevant non-major bleeding occurred in 4.3% of the patients in the apixaban group, as compared with 9.7% of those in the conventional-therapy group at 6 months [64]. Thus, it appears that the overall risk of bleed is high in cancer patients in the real-life setting as suggested by Khorana’s group [60] or in the more controlled setting of a randomized clinical trial [61–64].

### Radiation

Therapeutic radiation therapy (RT) is a cornerstone of cancer treatment. While RT significantly improves cancer outcomes and survival rates, chest radiation therapy (C-XRT) can also lead to both immediate and long-term radiation associated cardiac disease (RACD). As many as 81% of patients with RACD have valvular heart disease [26]. Left-sided valves are more commonly involved than right-sided valves, and changes can manifest as either stenosis or regurgitation. RT can damage valve leaflets, leading to fibrotic thickening, retraction and calcification. Perivalvular structures including the annulus, subvalvular apparatus, and aortomitral curtain are also prone to damage [27]. Thickening and calcification of the aortomitral curtain are hallmarks of prior heart irradiation, and the severity of these changes is linked to increased mortality risk [65]. Furthermore, with the multisystem impact of RT—including restrictive pulmonary disease, impaired wound healing, and recurrent pleural effusions—these patients often experience prolonged postoperative recovery [66]. Darby et. al demonstrated a linear increase in the risk of major coronary events by 7.4% per gray of radiation exposure to the heart, emphasizing the critical need to minimize cardiac dose during treatment to reduce long-term cardiovascular outcomes [67].

Prior chest radiation, particularly mantle fields for Hodgkin’s lymphoma, can render patients poor surgical candidates due to mediastinal fibrosis and calcification—making transcatheter approaches preferable when anatomically feasible. These considerations support the expanding role of TAVR and other minimally invasive approaches in this high-risk subgroup.

Management of these patients is complex. Valve replacement is generally preferred over repair due to the abnormal nature of irradiated valve tissue, which is prone to progressive fibrosis and calcification. Valvular intervention in severe RACD is challenging due to extensive cardiac involvement and high surgical risk. Long-term mortality rates are significantly higher in RACD patients—45% for single-valve and 61% for multi-valve surgery—compared to 13% and 17% in non-radiated patients [66]. Long-term outcomes for RACD patients requiring MV surgery are poor. A study of 146 patients with significant MV disease found a 5-year survival rate of only 55%, significantly lower than non-radiation-exposed patients. Older age, higher Society of Thoracic Surgeons (STS) scores, coronary disease, redo surgery, and female sex were associated with increased mortality risk [68]. Patients with a history of RT and severe AS face worse outcomes after SAVR, with 48% mortality at 6 years versus 7% in non-radiated patients [69]. Notably, standard risk assessment tools, such as the STS score, tend to underestimate the 30-day mortality risk in RT survivors. A study comparing TAVR and SAVR in RACD patients reported similar short-term survival, with TAVR showing a trend toward better 1- and 2-year survival rates (91% and 86%) compared to SAVR (86% and 80%). However, long-term TAVR outcomes remain undefined in this population [70].

A meta-analysis evaluating the outcomes of TAVR in cancer survivors with prior C-XRT revealed comparable 30-day mortality, safety, and efficacy rates between those with and without a history of C-XRT. However, patients in the C-XRT group demonstrated significantly higher rates of 1-year mortality and congestive heart failure exacerbations, underscoring the long-term challenges faced by this unique patient population [71]. It is unclear whether patients at low surgical risk may still benefit from SAVR. A practical approach, pending further evidence, is to prioritize TAVR for patients at intermediate or high surgical risk, provided there are no technical or anatomical considerations.

Patients with a history of radiation therapy face complex challenges, including the potential recurrence of their primary malignancy or the development of secondary cancers [72]. Compounding these issues may be further indications for C-XRT. These individuals are at heightened risk for accelerated structural valve deterioration (SVD) of bioprosthetic valves [73]. Studies have shown that average lifespan of bioprosthetic valve is almost half in patients treated with radiation.

Radiation-induced injury to valve tissue leads to progressive fibrosis and calcification of the leaflets, compromising their structural integrity and functionality. Van Rijswijk et al. demonstrated that high-dose radiation contributes to cellular damage and early-onset fibrotic aortic valve stenosis, indicating a dose-dependent relationship between radiation exposure and aortic valve fibrosis [74]. Ongoing research and advancements in transcatheter techniques are essential to optimize outcomes and enhance the quality of life for cancer survivors facing the dual challenges of malignancy and radiation-induced cardiac disease.

## Conclusions

Cancer has evolved into a chronic disease for many patients, thanks to advancements in screening and therapies. Yet, cardiovascular complications—including valvular heart disease, structural cardiac anomalies, ischemic disease, and a heightened risk of stroke—remain significant causes of morbidity and mortality in this population. Transcatheter interventions, such as TAVR, TMVr, LAAO, and PFO/ASD closure, have opened new frontiers for delivering effective therapy to individuals who might otherwise be deemed too high-risk for surgery. Although limited by underrepresentation of patients with cancer in clinical trials, existing evidence points to encouraging outcomes, such as improved symptom control, fewer perioperative complications, and the potential to continue cancer treatments with minimal interruption.

Nevertheless, unique challenges persist in cancer therapies such as chemotherapy and radiation may aggravate structural heart disease, accelerate atherosclerosis, or exacerbate bleeding risks, while the hypercoagulable state inherent in malignancy predisposes to both thrombosis and hemorrhage. The balance between minimizing procedural risk and controlling disease progression underscores the vital need for close collaboration among oncologists, cardiologists, interventionalists, and surgeons (Fig. 1). Future research should focus on refining patient selection criteria, optimizing timing of interventions, and developing individualized management protocols that integrate oncologic staging with cardiac risk stratification (Fig. 2). Through a dedicated and multidisciplinary approach, we can ensure that these interventions become not only feasible but also beneficial for the growing population of cancer survivors who deserve both extended life expectancy and improved quality of life.

## Key References

- Ahsan U, Naz S, Anum A, Unum A, Hamza RM, Qasim RM, et al. Outcomes and Adverse Effects of Transcatheter Aortic Valve Replacement (TAVR) in Cancer Patients: A Meta-Analysis. Cureus. 2024;16(11):e73442.
  o A meta-analysis of 15 studies which show no difference in all-cause mortality between patients with cancer and those without who undergo TAVR.
- Letteri F, Pracucci G, Saia V, et al. Endovascular Treatment in Patients With Acute Ischemic Stroke and Comorbid Cancer: Analysis of the Italian Registry of Endovascular Treatment in Acute Stroke. Stroke: Vascular and Interventional Neurology. 2023;3(3):e000423. 10.1161/SVIN.122.000423
  o Study that shows comparable outcomes after mechanical thrombectomy for stroke in patients with and without cancer.
- Bungo, B., et al., Better prediction of stroke in atrial fibrillation with incorporation of cancer in CHA2DS2VASC score: CCHA2DS2VASC score. Int J Cardiol Heart Vasc, 2022. 41: p. 101,072.
  o An updated risk scoring system based on the CHA2DS2VASC score that adds cancer as a risk factor for VTE.

## Data Availability

No datasets were generated or analyzed during the study.
